# Prediction of chronological age and its applications in forensic casework: methods, current practices, and future perspectives

**DOI:** 10.1093/fsr/owad021

**Published:** 2023-06-30

**Authors:** Mie Rath Refn, Marie-Louise Kampmann, Niels Morling, Jacob Tfelt-Hansen, Claus Børsting, Vania Pereira

**Affiliations:** Section of Forensic Genetics, Department of Forensic Medicine, Faculty of Health and Medical Sciences, University of Copenhagen, Copenhagen, Denmark; Section of Forensic Genetics, Department of Forensic Medicine, Faculty of Health and Medical Sciences, University of Copenhagen, Copenhagen, Denmark; Section of Forensic Genetics, Department of Forensic Medicine, Faculty of Health and Medical Sciences, University of Copenhagen, Copenhagen, Denmark; Section of Forensic Genetics, Department of Forensic Medicine, Faculty of Health and Medical Sciences, University of Copenhagen, Copenhagen, Denmark; The Department of Cardiology, The Heart Centre, Copenhagen University Hospital, Rigshospitalet, Copenhagen , Denmark; Section of Forensic Genetics, Department of Forensic Medicine, Faculty of Health and Medical Sciences, University of Copenhagen, Copenhagen, Denmark; Section of Forensic Genetics, Department of Forensic Medicine, Faculty of Health and Medical Sciences, University of Copenhagen, Copenhagen, Denmark

**Keywords:** forensic genetics, age estimation, molecular methods, DNA methylation

## Abstract

Estimating an individual’s age can be relevant in several areas primarily related to the clinical and forensic fields. In the latter, estimation of an individual’s chronological age from biological material left by the perpetrator at a crime scene may provide helpful information for police investigation. Estimation of age is also beneficial in immigration cases, where age can affect the person’s protection status under the law, or in disaster victim identification to narrow the list of potential missing persons. In the last decade, research has focused on establishing new approaches for age prediction in the forensic field. From the first forensic age estimations based on morphological inspections of macroscopic changes in bone and teeth, the focus has shifted to molecular methods for age estimation. These methods allow the use of samples from human biological material that does not contain morphological age features and can, in theory, be investigated in traces containing only small amounts of biological material. Molecular methods involving DNA analyses are the primary choice and estimation of DNA methylation levels at specific sites in the genome is the most promising tool. This review aims to provide an overview of the status of forensic age prediction using molecular methods, with particular focus in DNA methylation. The frequent challenges that impact forensic age prediction model development will be addressed, together with the importance of validation efforts within the forensic community.

## Introduction

Ageing is characterized by a progressive decline of biological function resulting in deterioration, frailty, and, eventually, death [1, 2]. Nine major cellular and molecular hallmarks have been proposed to contribute to the process of ageing each of which explains one or more aspects of the biological basis of ageing. These were extensively reviewed in López-Otín et al. [2]. They include genomic instability, telomere attrition, epigenetic alterations, loss of proteostasis, deregulated nutrient sensing, mitochondrial dysfunction, cellular senescence, stem cell exhaustion, and altered intercellular communication. These factors are interconnected, and their relationship is intricate [3].

An individual’s age is usually assessed by chronological age, defined as the period elapsed since birth to a specific point [4]. But age also reflects the biological changes that occurred within that period. Although chronological age can be used as a proxy for biological age, the rate at which age-dependent biological changes occur differs among individuals. Hence, biological age describes the general condition of an individual at a particular chronological age and may thus differ from the chronological age [4]. As such, it has been shown that higher biological age is associated with age-dependent diseases such as atrial fibrillation [5]. While the definition of chronological age is somewhat trivial, the biological age is harder to define, and sometimes, chronological and biological ages are used interchangeably in the literature. Estimating age can be relevant in several areas primarily related to the clinical and forensic fields. As this review focuses on forensic age estimation, the term “age” refers to chronological age rather than biological age hereafter.

Forensic genetic casework is related to three main areas: analysis of trace samples, individual identification, and relationship testing (paternity cases or other kinship cases). The biological material left by the perpetrator at a crime scene, e.g. bloodstains, semen, or hair, can be used for identification. The DNA profile obtained from the trace sample is compared to the DNA profile of a suspect or profiles in the national DNA database. However, in situations with no suspect and no DNA match in the crime DNA database, additional intelligence information such as biogeographical ancestry [6, 7] and externally visible characteristics (EVCs) (hair, eye, and skin colour) [7] inferred from the DNA can provide further investigative leads. In this context, age estimation can be particularly relevant to direct the police investigation and even provide information on age-related phenotypic traits such as hair colour and baldness.

Estimation of age is also beneficial in other forensic contexts. In immigration cases, when the age of young asylum seekers cannot be proven by valid identification documents, age assessment is usually performed. In these cases, the estimated age might influence the outcome of the asylum process since an individual’s age affects the person’s protection status under the law [8]. In disaster victim identification (DVI), estimation of age from biological material can also serve as a screening tool to narrow the list of potential missing persons. Age estimation is also an important aspect of forensic anthropology, where age-at-death often serves as a key feature in identifying skeletal remains and excluding particular missing persons [9]. Another potential application of age estimation is investigative genetic genealogy. Genetic information from direct-to-consumer companies performing genealogical investigations can be used to search for distant relatives of potential perpetrators through DNA profiles generated from samples recovered in criminal investigations. Knowing an individual’s age can help determine what generation to investigate when searching for the perpetrator among distant relatives.

## Age estimation methods in forensic casework

The first forensic age estimations were based on morphological inspections of macroscopic changes in bone and teeth [10]. Morphological methods for age estimation are still in use today, and different approaches are used based on the biological material available. These relate to post-adolescent bone and dental maturation and/or degeneration [11–13]. One of the major drawbacks of these traditional macroscopic forensic age estimation methods is the scope of their applicability. These methods require biological material with morphological age features, which limits the tissues suitable for investigation in the context of a crime scene. Moreover, damaging X-radiation might raise ethical issues when applied to young asylum seekers.

In this regard, several methods of molecular age estimation have been proposed based on the molecular and biochemical changes that occur within the cell during ageing. These methods may be applied to samples from human biological material that do not contain morphological age features and can, in theory, be used on crime scene traces containing only small amounts of biological material. Although specific proteins and RNA-based methods have been described [14–16], molecular methods involving DNA analyses are the most promising ones. DNA is a relatively stable molecule even in biological samples such as dried bloodstains, which makes DNA a suitable molecule for forensic age estimation [17]. Four groups of molecular biomarkers have been proposed for age estimation: mitochondrial DNA alterations, shortening of telomeres, signal-joint T-cell receptor excision circles, and DNA methylation. From these methods, changes in the pattern of DNA methylation have shown the greatest potential for estimating age in a forensic context [18].

### DNA methylation

DNA methylation is one of the best-understood epigenetic modifications in the genome [19, 20]. The most common form of DNA methylation is a methyl group on the fifth carbon of cytosine (5meC). This epigenetic modification predominantly occurs at cytosines (C) that are followed by guanines (G). This is referred to as CpG loci, where the p stands for the phosphodiester bond between the two nucleotides [21]. Approximately, 70% of the CpG dinucleotides in the human genome are methylated [22]. CpG sites are unevenly distributed throughout the genome, with most of the genome being CpG-poor. CpG sites occur in higher density in specific genomic regions called CpG islands, often located within or near promoter regions of genes [23]. Here, the methylation status of the CpGs influences gene expression: unmethylated sites are typically associated with active gene expression, while methylation of CpGs often leads to decreased transcription [20].

DNA methylation is dynamic, and DNA methylation levels change throughout life. CpG sites that become hypermethylated with age tend to be located within CpG islands, and as the CpG islands are often located within promoter regions, transcription of many genes is reduced with increased age [24]. In contrast, non-island CpGs loose methylation with age [24]. Most CpGs are located outside CpG islands, leading to an overall decrease in methylation levels with age. Early studies hypothesized that the gradual loss of methylation over time was due to the accumulation of errors during cell replication, where the DNA methylation was not preserved [25]. However, these errors are unsystematic, which means that the CpG sites that undergo stochastic changes over time might not be the same in every individual. Consequently, an increase in interindividual variability is observed with age [26]. This phenomenon is known as epigenetic drift. Today, epigenetic drift covers both the stochastic drift in DNA methylation and differences induced by the environment (e.g. differences in smoking habits, alcohol consumption, and diet) [27–29].

Even though epigenetic drift is a known and somewhat unpredictable phenomenon, specific CpGs have been identified, where the changes in DNA methylation levels with age are consistent across individuals. The DNA methylation of these CpGs has been used to construct models for predicting the chronological age [28, 30–32]. This phenomenon is known as the epigenetic clock. Epigenetic clock CpGs are mostly found within CpG islands, whereas non-island CpGs tend to be more affected by epigenetic drift. Consequently, age-associated CpGs predominantly gain methylation with age (called hypermethylation) although age-associated decreases in methylation (called hypomethylation) have also been observed [33, 34].

Many of the early studies on age-correlated DNA methylation were based on genome-wide examinations. Most often, these investigations were used to screen and select relevant age-correlated CpG sites, and thus, the majority of the most promising CpG sites for age determination were discovered through these investigations [33, 35, 36]. The most common approaches are whole-genome bisulphite sequencing (WGBS), reduced-representation bisulphite sequencing (RRBS), and microarray-based methods (for a detailed review of the methods, see Yong et al. [37]).

To this day, more than 100 CpGs in more than 50 genomic regions have been identified and applied for age-prediction in a forensic context. Table 1 presents a summary of 12 genes with age-associated CpGs included in forensic age-prediction models, the tissues examined, the ageing patterns of the CpGs, and the references from the literature.

**Table 1:** Age-associated candidate loci, CpGs described, tissues investigated, aging pattern, and references from the literature.

Gene^a^	CpGs^b^	Tissue	Ageing pattern	Reference
*ASPA*	cg02228185	Blood, buccal swabs, bones	Hypomethylation	[32, 40–42, 50, 64, 74, 76, 93]
*MIR29B2CHG*	chr1:207823672, cg10501210, chr1:207823681, chr1:207823702, chr1:207823705, chr1:207823715, chr1:207823723	Blood, saliva, buccal swabs, hair follicles	Hypomethylation	[47, 52, 56, 61, 63, 64, 68, 69, 71, 73, 76, 85, 90, 94–96]
*CCDC102B*	chr18:68722210, cg19283806	Blood	Hypomethylation	[49, 61, 63, 64, 73, 93–95]
*EDARADD*	chr1:236394441, cg09809672, chr1:236394371, chr1:236557695	Blood, saliva, buccal swabs, dental tissue	Hypomethylation	[40, 41, 57, 73, 76, 89, 90, 93]
*ELOVL2*	chr6:11044585, chr6:11044587, chr6:11044590, chr6:11044625, chr6:11044628, chr6:11044631, chr6:11044634, chr6:11044640, chr6:11044642, cg16867657, chr6:11044647, cg24724428, cg21572722	Blood, saliva, buccal swabs, dental tissue, hair follicles, bones	Hypermethylation	[18, 40, 41, 47–49, 52, 54, 56, 58, 59, 61, 63, 64, 68, 69, 71, 73, 74, 76, 78, 89, 90, 94–97]
*FHL2*	cg06639320, chr2:105399288, chr2:105399291, chr2:105399300, chr2:105399316	Blood, saliva, buccal swabs	Hypermethylation	[47, 52, 56, 64, 68, 71, 73, 76, 89, 90, 93–95]
*ITGA2B*	chr17:44390358, cg25809905, chr17:44390374, chr17:44390412	Blood, buccal swabs	Hypomethylation	[32, 42, 50, 66, 74, 93]
*KLF14*	chr7:130733453, cg14361627, chr7:130734357, chr7:130734372, chr7:130734375, chr7:130733483, cg04528819	Blood, saliva, buccal swabs, hair follicles, bones	Hypermethylation	[47, 51, 52, 54, 56, 57, 59, 69–71, 76, 78, 80, 87, 93, 95]
*NOX4*	cg06979108	Semen	Hypermethylation	[53, 55, 72, 75]
*PDE4C*	chr19:18233078, cg17861230, chr19:18233105, chr19:18233127, cg01481989, chr19:18233133, chr19:18233193	Blood, buccal, dental tissue, hair follicles, bones	Hypermethylation	[40, 41, 50, 57, 61, 64, 66, 69, 74, 76, 89, 90]
*TRIM59*	chr3:160450174, cg07553761, chr3:160450192, chr3:160450199, cg15618978	Blood, saliva, buccal swabs	Hypermethylation	[47, 52, 54, 56, 59, 61, 68, 71, 73, 76, 78, 95]

a The loci included in the table have shown correlations between DNA methylation levels and chronological age and have been incorporated into prediction models in at least four published studies.

b CRCh38 positions are shown for CpGs without cg number.

## Analysis of DNA methylation—forensically relevant methods

**Figure 1:**
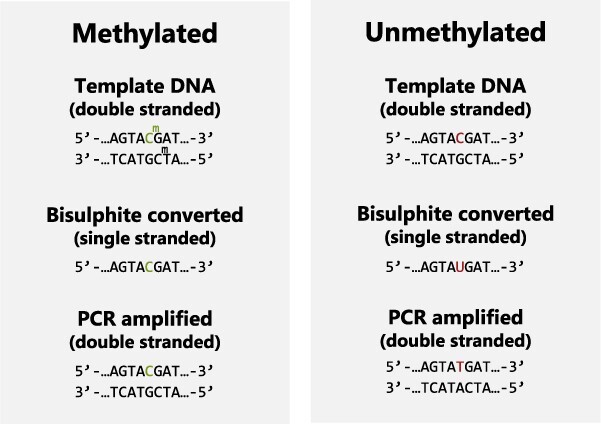
Bisulphite conversion of methylated and unmethylated citosines.

Most methods used for detection of DNA methylation require bisulphite conversion and PCR amplification of the DNA. Sodium bisulphite deaminates unmethylated cytosines (C) and converts them into uracils (U), whereas methylated cytosines are protected from deamination and remain unaltered (Figure 1). PCR amplification of the bisulphite treated DNA using dNTPs (dATP, dCTP, dGTP, and dTTP) changes uracils to thymines (T). Thus, the methylation status can be determined by the proportion of PCR molecules with C or T in a specific CpG position, which resembles genotyping of single nucleotide polymorphisms (SNPs). One of the drawbacks of bisulphite treatment is the considerable loss and fragmentation of the DNA, which reduces the number of molecules that can serve as a template for the downstream PCR analysis [38]. Consequently, high amounts of input DNA (∼200–500 ng) are recommended for the bisulphite treatment, which is rarely compatible with the amount recovered from crime scenes [39].

DNA is treated with sodium bisulphite. Unmethylated cytosines are converted to uracils, whereas methylated cytosines stay unaltered. During subsequent PCR amplification the uracils are converted to thymines, thus, the methylation status of the original DNA molecule can be interpreted as a cytosine or thymine after the PCR.

**Figure 2:**
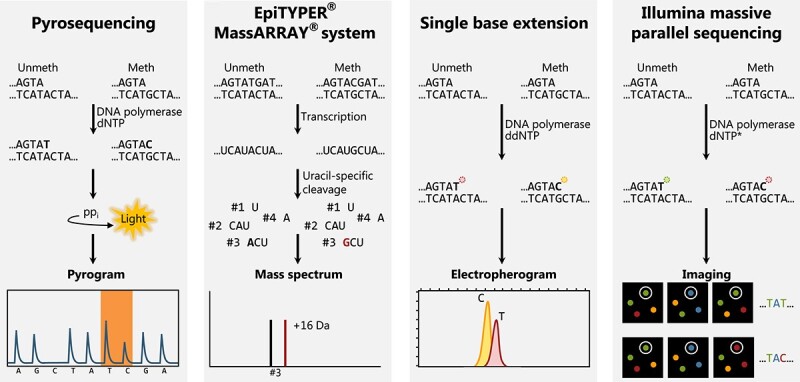
Technologies for determination of DNA methylation in forensic genetics.

Current forensic methods for determining DNA methylation rely on high-sensitivity/high-throughput techniques, although there is no standard technology adopted. Several technologies used for standard SNP genotyping have been applied to studies of DNA methylation, including pyrosequencing, single base extension (SBE), mass spectrometry, and massively parallel sequencing (MPS) (see Table 2 and Figure 2).

**Table 2:** Technologies for determination of DNA methylation in forensic genetics and their main characteristics in regard to DNA methylation analysis.

Technique	Detection platform	Length of analyzed region (bp)	Target detection by platform	Methylation status
Pyrosequencing	PyroMark	<100	One target region per analysis	All CpGs in the target region
Base-specific cleavage of ssRNAs^a^	MALDI-TOF mass spectrometer	100–600	One target region per analysis	All CpGs in the target region
SBE	Capillary electrophoresis	1	Multiplexing of target regions is possible	One CpG per SBE primer
MPS	MPS platform	<300	High level of multiplexing of target regions is possible	All CpGs in target region

SBE: single base extension; MPS: massively parallel sequencing; MALDI-TOF: matrix-assisted laser desorption-ionization time of flight.

a EpiTYPER® MassARRAY® system.

The input for all methods is PCR products from amplification of bisulphite-treated DNA, as seen in Figure 2. (i) Pyrosequencing: each of the four nucleotides are added sequentially to the sequencing reaction. As a nucleotide is incorporated into the strand, pyrophosphate is released and used by an enzymatic cascade leading to the generation of light. The burst of light is detected by a camera. The level of methylation of a given CpG can be interpreted as the ratio of incorporated C or Ts at the CpG locus and would be approximately 33% with the given pyrogram. (ii) EpiTYPER® MassARRAY® system: after PCR amplification, *in vitro* transcription of the PCR products is carried out. Methylated cytosines are transcribed to guanine, while unmethylated cytosines, converted to uracils after bisulphite conversion and turned into thymine after PCR amplification, are transcribed to adenines. Transcription is followed by uracil-specific-cleavage of the RNA and the RNA fragments are analyzed by MALDI-TOF MS. Adenines are 16 Da lighter than guanines resulting is two peaks in the mass spectrum. The level of methylation of a given CpG is determined by the peak heights of the two RNA fragments in the mass spectrum. In the given mass spectrum, the fragment “CAU” would have the same mass as the unmethylated fragment “ACU” of the given CpG thus, the level of methylation for the CpG would be approximately 66% as “CAU” takes up two-thirds of the peak height of the black peak. (iii) SBE: an SBE primer, complimentary to the region adjacent to the CpG of interest, anneals to the single stranded PCR product. The primer is extended by one fluorescently labelled dideoxy nucleotide complimentary to the nucleotide present at the interrogated position. The identity of the incorporated nucleotide can be interpreted from the colour of the fluorescence detected by capillary electrophoresis. Here, the level of methylation is given by the peak height ratio between the methylated and the unmethylated allele and would be approximately 60% for the given GpG. (iv) Illumina sequencing-by-synthesis: DNA synthesis is performed in the presence of all four nucleotides with different fluorescently labelled reversible terminators (marked by an asterisk). Once the nucleotide is incorporated, the fluorescence is detected by a camera. The terminator is chemically removed allowing incorporation of the next nucleotide into the growing strand. The level of methylation of a given CpG can be interpreted as the number of reads containing a cytosine *versus* a thymine at the CpG position. ddNTP = dideoxynucleotide triphosphates, dNTP = deoxynucleotide triphosphate, pp_i_ = pyrophosphate.

Early models proposed for forensic prediction of age relied on pyrosequencing and the PyroMark platform [32, 40–42]. Pyrosequencing is a sequencing-by-synthesis method based on the release of pyrophosphate and light detection, whenever a nucleotide is incorporated onto the growing DNA strand [43]. The level of methylation of a CpG can be interpreted as the ratio of incorporated C or Ts at the CpG locus and reported as percentage of methylation [44]. The usual read length is less than 40 nucleotides; however, read lengths of up to 100 bp can be achieved [45]. Thus, only closely positioned CpG sites may be analyzed on the same fragment. One of the significant disadvantages of pyrosequencing is the low multiplexing capacity of the PyroMark, where only one amplicon can be sequenced at a time. This increases the amount of DNA input needed to analyze CpG sites from several regions of interest, as a reaction would need to be carried out for each region of interest. However, multiplex amplification strategies for DNA methylation analysis through pyrosequencing are emerging [46]. Despite this limitation, pyrosequencing is still the most used technique for quantifying DNA methylation [47–59].

The EpiTYPER® MassARRAY® system is a MALDI-TOF mass spectrometry-based detection method that relies on the mass difference between transcribed RNA fragments [60]. Several age-prediction models have been developed based on this system [18, 61–66]. After bisulphite treatment and PCR amplification with tagged PCR primers, the PCR products are transcribed in the reverse direction, and the RNA is cleaved after every uracil residue in the RNA. The RNA fragments are analyzed by MALDI-TOF mass spectrometry, and the mass of each RNA fragment is measured. On the reverse strand, cytosines converted to uracils by the bisulphite treatment are adenines, whereas methylated cytosines are guanines. The sequence change from guanine to adenine results in a mass shift of 16 Da [60]. The level of methylation of a given CpG locus is determined by the peak heights of the two RNA fragments in the mass spectrum. Closely positioned CpGs may be present on the same RNA fragment, and the methylation levels of these can only be determined indirectly. Furthermore, only one transcribed molecule can be investigated in each reaction.

Other techniques with higher multiplexing capability have also been proposed for detection of DNA methylation in forensic genetics. SBE is a well-known method for SNP typing that has been used for standard forensic casework [67], which makes the implementation of the method for age determination straightforward. DNA methylation can be detected using the same method, and several studies have used this technology for age-prediction [68–72]. After bisulphite treatment and PCR amplification, the SBE reaction is performed as consecutive cycles of denaturation of the double-stranded DNA, annealing of the SBE primers to the PCR products, and single base extension. The SBE primer anneals to the single-stranded PCR product immediately upstream of the CpG position. The DNA polymerase adds a fluorescently labelled dideoxyribonucleotide (ddNTP), complementary to the nucleotide in the CpG position, to the SBE primer. The SBE product is then analyzed by capillary electrophoresis, where the identity of the incorporated nucleotide can be interpreted from the colour of the fluorescence. Here, the level of methylation of a CpG is given by the peak height ratio between the methylated and the unmethylated allele. The downside of the method is that only CpGs for which SBE primers were designed can be investigated. Therefore, the methylation status of neighbouring CpGs remains unknown unless SBE primers for these loci are also designed and included in the multiplex SBE reaction.

In the last couple of years, MPS has gained interest in forensic age determination, and most of the recent prediction models employ PCR-MPS methods [73–76]. MPS covers multiple platforms for high-throughput sequencing that use a variety of different sequencing chemistries (reviewed in Moorthie et al. 2011 [77]). For MPS of bisulphite-converted DNA, the MiSeq system has been the platform of choice [73–76, 78]. MiSeq uses sequencing-by-synthesis chemistry, where all four nucleotides with different fluorescently labelled reversible terminators are added to the reaction. The incorporated nucleotide is detected *via* the fluorescent label, and DNA synthesis is resumed once the terminator is chemically removed [79]. The level of methylation of each position is given by the proportion of reads with C or T alleles. MPS can determine the methylation status of neighbouring CpG loci on the same amplicon. Furthermore, with the incorporation of molecular barcodes, several individuals can be sequenced simultaneously, which decreases the cost per experiment. Nevertheless, the price might be prohibitive for implementing the method in forensic laboratories. Urgent criminal casework could become expensive, because it may not always be possible to maximize the sequencing run by investigating many samples in one run.

Due to the quantitative nature of DNA methylation assessment, the methylation levels found in a CpG site might not be identical or similar to those detected using other techniques or platforms [61, 80]. Therefore, the models are often platform-specific, and the pros and cons of using each platform should be weighted. Attempts have been made to apply models from one platform to data generated with another; however, usually large differences in the DNA methylation are observed resulting in large prediction errors. To some extent, these errors can be reduced by applying data correction [61, 80, 81].

## Developing an age-prediction model for forensic casework—challenges and status

The first forensically relevant age-prediction model based on DNA methylation was proposed by Weidner et al. in 2014 [32]. The number of reported models has continued to grow since then, and to this day, there are more than 40 studies on DNA methylation-based age estimation in a forensic context, most of which were published from 2017 to 2022. An overview of the published studies, including a summary of the main characteristics—technology used, cohort and tissue types investigated, CpGs covered, and prediction accuracy of the models is presented in Supplementary Table S1 (last revised December 2022).

The rationale behind model development is to use the methylation levels of age-informative CpGs and the corresponding chronological age of the studied individuals to build a model for predicting the age of an individual with unknown age. The performance of a model is evaluated by the difference between the predicted and chronological age of the tested individuals. The performance is often presented as the mean absolute deviation (MAD) or mean absolute error (MAE), used interchangeably, although several other metrics have been described. Throughout this review, the MAD will be used whether or not MAD or MAE were used in the original paper.

In the following sections, we will describe the most important factors influencing the detection and distribution of DNA methylation in the human genome, their consequences, and the status of DNA methylation age-prediction models.

### DNA requirements

DNA methylation analysis requires higher amounts of DNA than what is typically available in forensic trace samples. This requirement is mainly due to bisulphite treatment greatly degrading the DNA (DNA recovery down to ~30% of input DNA) [38, 82]. Moreover, loss in precision of the methylation quantification is observed when the DNA input amount is low, as a small percentage differences in methylation cannot be quantified from a low number of cells [83]. Further, the quantification of DNA methylation is also more impacted by stochastic effects when the DNA input amount is low [83].

It has been proposed that a threshold of minimum 1 000 sequencing reads are required to retain accuracy in the provided methylation levels [84]. However, a high number of reads cannot compensate for deviations caused by stochastic effects due to low-input amounts of DNA [83]. Furthermore, studies have suggested that 10–20 ng of DNA template is required for the PCR step (after bisulphite conversion) to obtain a reliable methylation quantification [48, 83]. In agreement with this, Woźniak et al. [76] observed robust quantifications down to 20 ng DNA input (8.8–11.8 ng DNA template after bisulphite conversion), while lower input amounts resulted in increased variability in methylation levels when evaluating the sensitivity of the DNA methylation assay. Similarly, Ambroa-Conde et al. [85] observed significant deviations in DNA methylation values when the DNA input was 1 ng and recommended a minimum of 10 ng of genomic DNA for their assay. Developing good age-prediction models using lower DNA inputs is one requirement for implementing age estimations in forensic casework. Aliferi et al. [73] reported a prediction model that relied on sites with strong correlations between methylation levels and chronological age and had an extensive methylation range over the human lifespan (overall methylation range above 60%). Using this marker selection strategy, the model could retain the accuracy of the age-prediction down to an input of 5 ng of DNA (~1 ng input in the PCR). This input is much lower than any other age-prediction model and close to the input levels used for standard genotyping methods.

### Forensically relevant tissues and body fluids

Analysis of DNA from whole blood or saliva samples is convenient when developing age-prediction models, since these tissues are easily accessible. Therefore, most of the forensic age estimation models are based on DNA methylation in blood [18, 32, 41, 42, 47–49, 51, 52, 54, 56, 58, 59, 61–64, 66, 68, 71, 73, 74, 76, 78, 86–97]. Some studies have focused on DNA from buccal swabs [40, 50, 57, 71, 76] and saliva [51, 70, 71, 80, 98]. These body fluids are also relevant in a forensic context, both for crime and immigration casework. Other tissues and body fluids such as dental tissue [41, 65], hair follicles [69], bones [76], and semen [53, 55, 72, 75, 88] have been used to develop age-prediction models as well.

Samples used to develop age-prediction models are usually high-quality single-source tissue samples or body fluids, while forensic casework samples are often low-quality traces from cigarette butts, swabs with blood or seminal stains, vaginal swabs, etc. Furthermore, the DNA is often partly degraded, and samples often contain DNA from multiple donors. For implementation into standard forensic casework, age-prediction models must be assessed in the context of the actual forensic samples. Moreover, the stability of the DNA methylation over more extended periods needs to be considered. Hamano and co-workers [98] tested their age-prediction model for saliva on DNA extracted from seven cigarette butts to simulate samples from an actual crime scene. They observed a decrease in the prediction accuracy when validating the model on the cigarettes compared to validation in an independent dataset of 50 saliva samples (MAD of 6.25 years compared to 7.65 years). Multiple models developed for blood have been tested in bloodstains with little or no difference in the prediction accuracy, regardless of the time of storage, indicating the usefulness of blood-based predictions for age estimation in bloodstains [42, 48, 54]. In concordance, Han et al. [95] performed an age prediction on DNA extracted from a bloodstain at a crime scene using two prediction models developed for blood (*ELOVL2, FHL2, MIR29B2CHG, CCDC102B, KLF14, SYNE2, TRIM59*, and *cg26947034*). The predicted age was 38.27 and 38.59 years, and after the case was solved, it was confirmed the suspect was indeed 38 years old. Lee et al. [99] observed a slight decrease in the accuracy of their age-prediction model for semen collected from crime scenes compared to fresh semen samples (MAD of 5.2 years compared to 4.8 years). The authors explained the decrease in accuracy as a likely influence of various factors such as storage conditions, the presence of other body fluids, and external contamination in the trance samples.

Most prediction models developed to date are tissue-specific, meaning the source tissue of a trace sample must be known or determined beforehand. Attempts to develop DNA methylation-based forensic tissue identification models have been carried out (for a detailed review, see Kader et al. [100]), which open up for combining tissue identification and age correlation CpGs into one model. This idea has been implemented for models developed for buccal swabs. Buccal swabs are highly heterogeneous and consist of a mixture of buccal epithelial cells and leukocytes. Furthermore, the composition of the mixture can vary considerably between samples [101]. Eipel et al. [50] tried to overcome this problem by introducing tissue-specific CpG sites for buccal epithelial cells (using *CD6* and *SERPINB5*) into their model. In combination with the age-associated CpGs, they observed age-predictions with MADs of 5.09 and 5.12 years in two independent validation sets. Similarly, Hong et al. [70] observed improved age-prediction when incorporating a cell-type-specific CpG (*PTPN7*) into their saliva-based age-prediction model (using *KLF14, TSSK6, TBR1, CNGA3, SLC12A5,* and *SST*) suggesting that determination of the cellar composition can improve age-predictions. Ambroa-Conde et al. [85] developed an age prediction model for both buccal swabs and saliva samples comprising seven CpGs (cg10501210, *LHFPL4, ELOVL2, PDE4C, HOXC4, OTUD7A*, and *EDARADD*). Opposed to the studies mentioned above, the addition of the tissue of origin as a variable did not significantly improve the age-predictions (MAD of 3.84 years compared to 3.78 years).

The age-prediction models developed for blood seem to have higher accuracy than those developed for other tissues. This is partly because the age correlations of most of the CpGs included in prediction models were originally found in studies investigating blood samples. This suggests that the best markers for accurate age-prediction are tissue specific. However, studies have found consistent age-association of CpGs among different tissue types. Naue et al. [102] investigated the correlation of existing blood markers in brain, bone, muscle, and buccal swabs. They identified seven CpG loci showing age-correlated DNA methylation levels in all tissues. Interestingly, it was not the same CpG positions in the loci that were correlated with age in the different tissues. However, as their study was a proof-of-concept study based on only 29 samples, more samples of each tissue are needed to fully investigate the tissue independency of the CpGs.

Few studies have tried to validate whether models developed for one tissue can be adapted to other tissues. Eipel et al. [50] applied the blood-based 3-CpG-model initially developed by Weidner et al. [32] to buccal swab samples and observed an average overestimation of 14.6 years in their samples. This effect seemed consistent rather than random. Therefore, the model was re-trained on buccal swab samples, and the result was a lower MAD of 4.3 years. In another study, Woźniak et al. [76] developed a multiplex assay for age prediction in blood, buccal cells, and bones. The assay comprised a multiplex of 44 CpG sites within eight age-associated genes (*ELOVL2, MIR29B2CHG, TRIM59, KLF14, FHL2, EDARADD, PDE4C,* and *ASPA*). Three prediction models were developed consisting of five to six CpGs depending on the tissue. All three models showed high prediction in the respective tissue (MAD of 3.2 years in blood, 3.7 years in buccal cells, and 3.4 years in bones). However, the models were tissue-specific, and source tissue evaluations were needed before choosing between tissue-specific models. Jung et al. [71] proposed a multi-tissue (blood, saliva, and buccal swabs) age-prediction assay with five CpG sites (*ELOVL2, FHL2, KLF14, MIR29B2C,* and *TRIM59*). Separate age-prediction models for the three sample types showed MADs of 3.48 years in blood, 3.55 years in saliva, and 4.29 years in buccal swab samples, while a combined-tissue model showed a MAD of 3.84 years when applied on an independent validation set compromising all three tissue types. This prediction accuracy was comparable to those found in tissue-specific models.

### Population influence

Ancestry-related differences in age-associated DNA methylation patterns have yet to be investigated fully. Most studies of forensic age estimation have focused on either European or Asian populations (see Supplementary Table S1), and the influence of biogeographical origin on DNA methylation patterns has, with a few exceptions, rarely been examined.

The model proposed by Zbieć-Piekarska et al. [47] in 2015 comprised five CpGs located in the five loci *ELOVL2, MIR29B2CHG, TRIM59, KLF14,* and *FHL2* and was developed based on a Polish population. This model was later evaluated in Korean and Singaporean populations [52, 54]. Within the five loci, a total of 32 CpG positions were re-analyzed and re-trained to generate new models on the two Asian populations. The new models compromised three CpGs for the Singaporean individual (*TRIM59, KLF1*, and *ELOVL2*) and 5–6 CpGs for the Korean individuals (*KLF14, FHL2, ELOVL2, TRIM59*, and *MIR29B2CHG*). Hereafter, the prediction accuracies of the original and new models were examined. For the Korean individuals, the original prediction model worked well with similar prediction accuracy to Polish individuals (MAD of 4.18 years for Korean and 3.9 years for Polish validation samples), while a somewhat higher prediction error was observed in the Singaporean individuals (MAD of 4.8 years). Regarding the new models, there was an increase in the accuracy for the Korean individuals (MAD of 3.34/3.29 years), while for the Singaporean individuals, the accuracies of the new and old models were the same. Although this might indicate that the five proposed loci are not be the optimal markers in the Singaporean population, this approach could not distinguish, if the differences observed were due to technical differences between the laboratories.

Other studies have evaluated age-prediction models within the same laboratory with the same set-up. Fleckhaus et al. [103] analyzed the methylation at five CpG sites within *ASPA, ITGA2B, PDE4C,* and *ELOVL2* from two previously reported age-prediction models in population groups from the Middle East, West Africa, and Central Europe. An overall high similarity in the change of methylation with age for the investigated CpGs was observed, but statistically significantly lower age-prediction errors were observed for the Middle Eastern population compared to the Central European and West African populations for both models.

Thong et al. [59] analyzed CpGs within *ELOVL2, KLF14, TRIM59,* and *FHL2* in Singapore-based populations of Chinese, Malays, and Indians and established an age-prediction model using all three subpopulations. No statistically significant difference in prediction error was observed among the three subpopulations (*P* = 0.53). In the same study, the authors used DNA methylation data from Polish and French individuals previously reported by other studies [47, 104]. Compared to the Singaporean subpopulations, notable differences in the prediction accuracy were observed for the Polish and French individuals (*P* ≤ 0.003). However, these differences could be due to technical variations between laboratories (DNA extraction, bisulphite conversion, etc.).

### Influence by sex, lifestyle, and diseases

Despite being widely studied, contradicting results have been reported on how gender affects age-associated DNA methylation. Weidner et al. [32] and Zbiec-Piekarska et al. [47] found slight differences in the age-prediction accuracy between males and females. In both studies, the predicted age was higher in males than in females, and the prediction accuracies were higher for females. The sample size of both studies was small, and the differences were not statistically significant.

Most studies on age-prediction based on DNA methylation have failed to detect any effect of sex on DNA methylation [18, 41, 50, 64, 92, 93, 95]. The sensitivity towards sex may vary depending on the CpG sites selected, which can explain the contradicting results. Nevertheless, when building age-prediction models, selecting CpGs that show comparable methylation levels among the two sexes is important.

Exogenous factors, including disease status and lifestyle factors (alcohol intake, smoking habits, etc.), have also been shown to impact DNA methylation patterns [105, 106]. For a detailed review, see Koop et al. [107]. Excessive alcohol intake leads to premature ageing and is associated with an increased risk of mortality [108, 109]. The impact of alcohol consumption on age-associated DNA methylation is not straightforward. Studies have shown a dose-dependent effect of alcohol consumption on age-associated DNA methylation levels. Low or high alcohol consumption levels resulted in age acceleration, while intermediate levels of alcohol consumption seemed to have a decelerating effect [110, 111]. Weidner et al. [32] found that high alcohol consumption was associated with a slight overestimation of age and increased the estimation error of the model (*PDE4C, ASPA, ITGA2B*). Piniewska-Róg et al. [112] investigated the association between high alcohol consumption and the precision of the model proposed by Woźniak [76] (*ELOVL2*, *MIR29B2CHG*, *KLF14*, *FHL2*, *TRIM59*, *PDE4C*, *EDARADD*, and *ASPA*). The authors found that the mean predicted age of alcohol abusers was higher (1.4 years) compared to controls of the same age and sex, indicating slightly faster ageing in alcohol abusers. This acceleration was mainly associated with one CpG in *MIR29B2CHG* but did not affect the overall prediction accuracy of the age model.

Tobacco smoking is a leading cause of disease and premature death [113, 114]. While some studies have found an association between smoking and age acceleration [110, 115, 116], others have failed to detect any association [111]. As part of their model development, Hamano et al. [98] investigated whether smoking affected methylation of age-associated CpG sites and did not find statistically significant differences among non-smokers, former, or current smokers. Similarly, Eipel et al. [50] compared 22 smokers and 179 non-smokers and found no differences in methylation levels for their selection of CpG sites.

Diseases such as cancer, cardiovascular disease, and ageing-related diseases have been shown to alter the relationship between DNA methylation and age [117–120]. Spólnicka et al. [121] analyzed methylation levels in five CpGs from five of the most used genes in forensic age-prediction (*ELOVL2, MIR29B2CHG, KLF14, FHL2,* and *TRIM59*) in three groups of individuals with early or late-onset Alzheimer’s disease or Graves’ disease. The CpGs in *ELOVL2* and *MIR29B2CHG* did not show disease-related alterations, indicating robustness in their DNA methylation patterns. However, the CpGs in *TRIM59* showed hypermethylation in the group of early onset Alzheimer’s disease as well as Graves’ disease patients while the CpGs in *FHL2* showed hypomethylation in the latter. Similarly, Aliferi et al. [73] investigated the impact of schizophrenia, rheumatoid arthritis, frontal temporal dementia, and progressive supranuclear palsy on the methylation of the 11 CpGs included in their age-prediction mode. Still, they did not find differences in methylation in diseased individuals. Notably, two of the CpGs in their model came from the same loci (*FHL2* and *TRIM59*), as reported by Spólnicka et al. [121]. They found hypermethylation and decreased prediction accuracy for *TRIM59* in early-onset Alzheimer’s disease patients. While altered DNA methylation leading to decreased prediction accuracy was found for both *TRIM59* and *FHL2* in the group of Graves’ disease, indicating differences in how various diseases affect the methylation of age-correlated CpGs.

More research into the robustness of age-associated CpGs is needed to fully understand the impact of exogenous and endogenous factors on DNA methylation-based age estimation for implementation in forensic casework.

## Importance of validation

### Intra-laboratory validation

Once developed, an age-prediction model needs to be validated. Without proper validation, it is impossible to ascertain how well the model performs on data not used to build the model (unseen data). Model validation also helps identify issues such as overfitting, e.g. when a model contains too many variables. This results in a close fit to the data on which the model was built (training data) but poor performance on other data. Usually, the validation is carried out by testing the model’s prediction accuracy on a separate dataset (validation set). The reported error of the model can depend greatly on how the validation dataset was selected. Ideally, the validation dataset should be done on an external dataset. However, the validation dataset most often derives from randomly dividing the data into a training and a validation dataset. Most proposed forensic age-prediction models have been validated using this approach, and one of the risks is an overestimation of the performance [122]. Another commonly used validation method is cross-validation, where the original sample is randomly split into *k* equal-sized subsamples. One subsample serves as the training data, while the remaining samples serve as the validation data. This procedure is repeated *k* times until all subjects have been used to validate the model. The performance of the model is then presented as the average performance of the *k* repeats. Compared to the data-splitting approach, cross-validation may use a large part of the data to develop the model, which decreases the risk of overfitting [123].

Another consideration is the approach for statistical modelling. Most of the CpGs in current forensic age-prediction models were included based on a high correlation between their methylation levels and chronological age, usually observed as high Pearson or Spearman correlations. When using linear regression models, several assumptions are made, including homoscedasticity, which assumes equal variance in the age groups. Age-prediction models have struggled with fulfilling this criterion as the data have shown a non-constant error variance with age [47–49]. In practical terms, this is expressed as an increase in prediction errors with increased age. The decrease in the accuracy of linear regression models is also caused by the non-linear associations between age and methylation of some CpG sites. This is especially clear for CpGs in the gene *ELOVL2*, one of the most excessively used age-informative genes [41, 76, 86]. This raises doubt about linear models and whether they can capture all aspects of the complicated relationship between DNA methylation and age. Several approaches to account for non-linear patterns have been proposed, including power transformation before multivariate linear regression analysis and multiple quadratic regression [41, 76].

To better capture the complexity of DNA methylation with age, several machine-learning approaches have been used for model development [66, 73, 78, 80, 86, 87, 124]. Aliferi et al. [87] compared 17 statistical modelling approaches on the same set of 12 CpGs to select the optimal approach. Their findings suggested support vector machines as the most robust and accurate method. However, more research on larger datasets is needed to evaluate the performance of the different modelling approaches.

### Inter-laboratory validation

Inter-laboratory validations are essential for the universal implementation of age-prediction methods and models. Several recent studies have focused on validating published age-prediction models in other laboratories and with different setups. Pfeifer et al. [125] evaluated the performance of two previously published age-prediction models for blood and buccal swabs in independent validation sets. Both models were proposed by Bekaert et al. [40, 41] in two separate studies and were based on CpG sites in *ASPA, EDARADD, PDE4C,* and *ELOVL2*. Using the prediction models, they observed high prediction errors in two tissues (MADs of 9.84 and 8.32 years for blood and buccal swabs, respectively), which was much higher than the original study (MAD of 3.74 and 3.32 years, respectively). However, the experimental conditions, including input DNA, PCR methods, bisulphite kit, and analysis were different between the two studies, which the authors supposed could explain the differences observed. Nonetheless, inter-population viability can also play a role in the results observed. By re-training the prediction models on their data, Pfeifer et al. [125] statistically significantly increased the accuracy (MAD of 5.55 years and 4.65 years for blood and buccal swabs).

In another study, Daunay et al. [104] evaluated the performance of six age-prediction models using DNA methylation analysis by pyrosequencing and blood samples from 100 French individuals. The models included a single-locus model [48] and five multi-locus models [32, 41, 47, 49, 54]. The best performance was found for two of the multi-locus models (MAD of 4.5 years for Bekaert et al. [41] and 5.2 years for Thong et al. [54]). The observed MADs were much higher than the ones described in the original studies. The authors attributed the decrease in accuracy to variations during the implementation of the different pyrosequencing assays.

To truly test for inter-laboratory variability, the same samples must be evaluated in different laboratories to minimize variations. In line with this, five participating laboratories of the VISible Attributes through GEnomics (VISAGE) consortium validated a DNA methylation assay for semen [126]. The assay consisted of 13 CpG sites. The reproducible quantification of methylation levels and sensitivity using DNA methylation controls was tested. Furthermore, to evaluate the concordance between laboratories and mimic casework scenarios, DNA extracts and stains on FTA cards from two semen samples were also investigated. These samples showed robust methylation quantification in all laboratories. After validating the assay, a more streamlined assay combining only five of the loci in one multiplex (*SH2B2, EXOC3, CALR2, IFITM2,* and *NOX4*) was developed along with a model compromising sex CpGs from the five loci.

## Future perspectives and concluding remarks

It is evident from the number of recent studies that age estimation has gained much attention in the forensic field. Although DNA methylation-based forensic age-prediction seems to be the most promising method, one of the major drawbacks is the need for high DNA input amounts. Therefore, the field has recently focused on developing models that require less DNA input or applying methods that avoid bisulphite conversion. A bisulphite-free method for quantification of DNA methylation at base-level-resolution has been proposed [127]. This method combines two steps for converting 5mC and 5-hydroxymethylcytosine (5hmC) to dihydrouracil. Dihydrouracil is then converted to thymine during PCR amplification, enabling the methylation status to be interpreted as a C/T SNP. Compared to bisulphite conversion, this method is non-destructive and leaves more DNA molecules intact for the subsequent PCR step. Furthermore, this technique converts methylated cytosines into thymines. This is in contrast to bisulphite conversion assays that convert unmethylated cytosines to thymines. Most cytosines in the genome are unmethylated. Thus, by converting the methylated cytosines instead of unmethylated cytosines, the complexity of the DNA sequence is preserved, which improves mapping rates during sequence analysis. However, the stochastic effects occurring when the DNA input becomes low still apply to this method.

Studies have shown that tissue-specific age-prediction models allow for more accurate predictions than multi-tissue models. However, tissue-specific age-prediction models can only be used if the type of tissue is known. Considerations should be made as to whether it is more practical to use a less accurate age-prediction model that can be applied to all tissues or more accurate models for each tissue type.

Recently, a male-specific Y-chromosome-based age-prediction model was proposed [128]. The model could be useful for age determination of unknown male perpetrators in mixed male–female DNA samples, which are commonly obtained from physical or sexual assault cases. In the proof-of-principle study by Vidaki et al. [128], the authors investigated DNA methylation patterns of the Y-chromosome in blood samples. In addition to blood, semen is important for male-specific age-prediction in sexual assault cases. At the moment, age-prediction models for semen consist of CpGs located on the autosome and, thus, cannot be used if the semen is mixed with body fluid from the female donor in a sexual assault case. Research into the correlation between age and methylation of Y-chromosome CpGs in semen is therefore needed.

Ideally, an age-prediction model developed for forensic use should have a broad application and should not be influenced by the biogeographical origin of the donor of the biological tissue. While many of the same CpG sites are included in various prediction models, much work is still needed to understand the relationship between ancestry, DNA methylation patterns, and age-prediction. One solution could be to develop ancestry-specific prediction models. However, for forensic applications, this would require several models for all relevant populations and the additional determination of the ancestry of the donor before applying a model. To overcome this issue, Fleckhaus et al. [103] proposed to include ancestry-informative markers in the age-estimation models. Another solution could be the identification of CpGs with a neutral relationship to biogeographical origin. In this way, the models could be applied to casework without knowing the ancestry of the doner beforehand. A high level of within-population variability in DNA methylation has previously been shown [129]. Thus, a large number of samples are needed to identify ancestry-neutral CpGs, which has not been the case for the sample sizes of the studies carried out so far.

At the time of writing, the validation study by the VISAGE consortium is the first study of its kind [126]. Such inter-laboratory validation studies are essential to fully evaluate the technical variability in detecting DNA methylation levels and ensuring the selection of models that perform consistently. Further inter-laboratory exercises and collaborative initiatives, including a broader number of participants, are needed to fully address the issue of technical variability for each potential age-prediction model. This would also help to standardize protocols and methods before the implementation into forensic casework.

## Authors’ contributions

Mie Rath Refn wrote the first draft of the manuscript. All authors contributed to the final text and approved it. Niels Morling initial holds the position of Editorial Board Member for *Forensic Sciences Research* and is blinded from reviewing or making decisions for the manuscript.

## Compliance with ethical standard

This article does not contain any studies with human participants or animals performed by any of the authors.

## Disclosure statement

The authors report there are no competing interests to declare.

## Funding

The authors received no financial support for this study.

## Supplementary Material

Supplementary_Table_S1_owad021
